# Benderamide A, a Cyclic Depsipeptide from a Singapore Collection of Marine Cyanobacterium cf. *Lyngbya* sp.

**DOI:** 10.3390/md16110409

**Published:** 2018-10-26

**Authors:** Chi Ying Gary Ding, Ji Fa Marshall Ong, Hui Chin Goh, Cynthia R. Coffill, Lik Tong Tan

**Affiliations:** 1 Natural Sciences and Science Education, National Institute of Education, Nanyang Technological University, 1 Nanyang Walk, Singapore 637616, Singapore; ding_gary@yahoo.com.sg (C.Y.G.D.); marshall.ong@nie.edu.sg (J.F.M.O.); 2 p53 Laboratory, Agency for Science, Technology and Research (A*STAR), #06-06, Immunos, 8A Biomedical Grove, Singapore 138648, Singapore; hcgoh@p53lab.a-star.edu.sg (H.C.G.); crcoffill@p53Lab.a-star.edu.sg (C.R.C.)

**Keywords:** *Lyngbya* sp., marine cyanobacterium, cyclic depsipeptide, molecular networking

## Abstract

Benderamide A (**1**), a (*S)*-2,2-dimethyl-3-hydroxy-7-octynoic acid (*S*-Dhoya)-containing cyclic depsipeptide that belongs to the kulolide superfamily, was isolated from a Singapore collection of cf. *Lyngbya* sp. marine cyanobacterium using a bioassay-guided approach. While the planar structure of **1** was elucidated using a combination of 1D and 2D NMR experiments and MS analysis, the absolute configuration was subsequently achieved using the results obtained from Marfey’s analysis, comparative analysis of nuclear overhauser effect spectroscopy (NOESY) with the known compound **3**, and one dimensional-nuclear overhauser effect (1D-NOE). Although **1** did not display antiproliferative activity against MCF7 breast cancer cells, the presence of an Ala instead of Gly suggests a possible mechanistic pathway to explain the consequential decrease in cytotoxicity compared to the closely related **2**. In addition, results obtained from an LC–MS/MS-based molecular networking algorithm revealed two other closely related compounds encouraging further identification and isolation from the same marine cyanobacterium extract.

## 1. Introduction

Filamentous marine cyanobacteria continue to be a prolific source of bioactive secondary metabolites with intriguing structures that are products of biosynthetic pathways, such as nonribosomal peptides or hybrid of peptide-polyketide gene clusters [1]. Many of these metabolites are structurally related, therefore forming a superfamily. An example is the ‘kulolide superfamily’, which include kulolide and the viequeamides [2,3]. Members of the ‘kulolide superfamily’ are cyclic depsipeptides and are all biosynthesized from cyanobacteria. The first member, kulolide, was isolated and hence thought to be biosynthesized from the predatory cephalaspidean mollusk, *Philinopsis speciosa*. However, further investigations became necessary to establish the true biological origin of kulolide after observing all other members in this superfamily isolated exclusively from marine cyanobacteria. The predator–prey relationship established between the mollusk and marine cyanobacteria finally revealed the latter to be the true biosynthetic origin of kulolide [4]. This resulted in each member bearing structural hallmarks of marine cyanobacteria characterized by a β-hydroxyoctanoic acid-derived terminal alkynyl fatty acyl residue and a sequence of several amino acid and/or hydroxy acid residues. In addition, members of this superfamily can be further subcategorized into two groups based on one of the two terminal alkynyl fatty acyl residues: compounds that contain the 2,2-dimethyl-3-hydroxy-7-octynoic acid (Dhoya) moiety and molecules that contain the 3-hydroxy-2-methyl-7-octynoic acid (Hmoya) moiety. The structurally diverse members give rise to various bioactivities, including antitumor cytotoxicity (e.g., kulolides, cocosamides, and hantupeptins), antiparasitic activity (e.g., dudawalamides), and brine shrimp toxicity (e.g., yanucamides) to name a few [3,5,6,7,8].

In the recent review of marine natural products from Blunt et al., a majority of the newly discovered bioactive metabolites reported from marine cyanobacteria in the past five years (till 2016) are peptidic in nature [9]. This prompted our continued search for peptidic bioactive metabolites from local strains of filamentous marine cyanobacteria in Singapore. In this report, an organic extract of cf. *Lyngbya* sp., collected from St. John’s Island, displayed strong brine shrimp toxicity at 100 ppm. Subsequent purification using vacuum liquid chromatography and semipreparative HPLC of the extract afforded a new Dhoya-containing cyclic depsipeptide benderamide A (**1**). The planar structure of this compound was determined by extensive 1D and 2D NMR spectroscopic methods while its absolute configuration elucidated using nuclear overhauser effect spectroscopy (NOESY) as well as Marfey’s analysis by comparison with amino acid standards.

## 2. Results

An initial sample of cf. *Lyngbya* sp. (determined through microscopic examination of morphology, Figure S1, Supplementary Data) was collected in June 2016 from the intertidal areas of St. John’s Island, Singapore and preserved in 70% ethanol solution before work up. The sample was subsequently extracted exhaustively with 2:1 CH_2_Cl_2_−MeOH mixture. The crude extract obtained was fractionated with normal-phase silica vacuum flash chromatography based on a combination of hexanes, EtOAc and MeOH with increasing polarity, giving a total of nine subfractions (A–I). Each of the subfractions was subjected to the brine shrimp toxicity assay and the fraction eluted with 100% EtOAc (fraction G) demonstrated 92.6% toxicity at 100 ppm. This fraction was subsequently filtered using Sep-Pak C_18_ followed by a series of reversed-phase HPLC to yield benderamide A (**1**) (Figure S2, Supplementary Data).

### 2.1. Structural Elucidation of **1**

**Figure 1:**
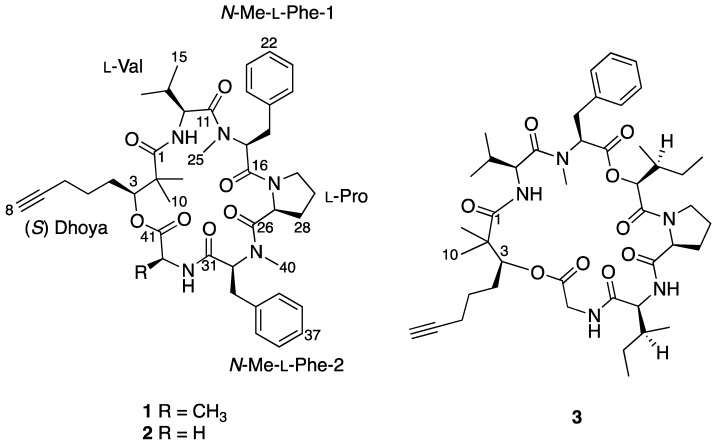
Complete chemical structures of benderamide A (**1**), cocosamide B (**2**), and pitipeptolide A (**3**).

Benderamide A (**1**) (Figure 1) was purified as an amorphous solid and positive ion HRESIMS analysis revealed its protonated adduct at *m*/*z* 756.4327, the molecular formula of **1** determined as C_43_H_57_N_5_O_7_ to account for 18 degrees of unsaturation. The peptidic nature of **1** was apparent from its ^1^H NMR spectrum and determination of its planar structure was based on detailed examination of various 1D and 2D NMR spectroscopic data. At least four amino acid residues were evident from two methylated H_3_ tertiary amide singlet signals at δ_H_ 3.59 and 2.79 as well as two secondary amide doublet signals at δ_H_ 9.03 and 5.70. The presence of at least two overlapping ^1^H spin systems at the aromatic region of δ_H_ 7.06–7.42 (10 Hs) was indicative of two phenyl-containing amino acids. Furthermore, the methine signal at δ_H_ 1.97 that resulted in a triplet with a small coupling constant (*J* = 2.5 Hz) suggested the presence of a terminal alkyne (Table 1). ^13^C NMR spectral data revealed six highly deshielded signals from δ_C_ 168.9–176.6, attributed to carbonyls of either ester or amide moieties, indicating the possibility of **1** having at least an ester group. The above characteristics of the compound eventually accounted for 16 out of 18 degrees of unsaturation.

**Figure 2:**
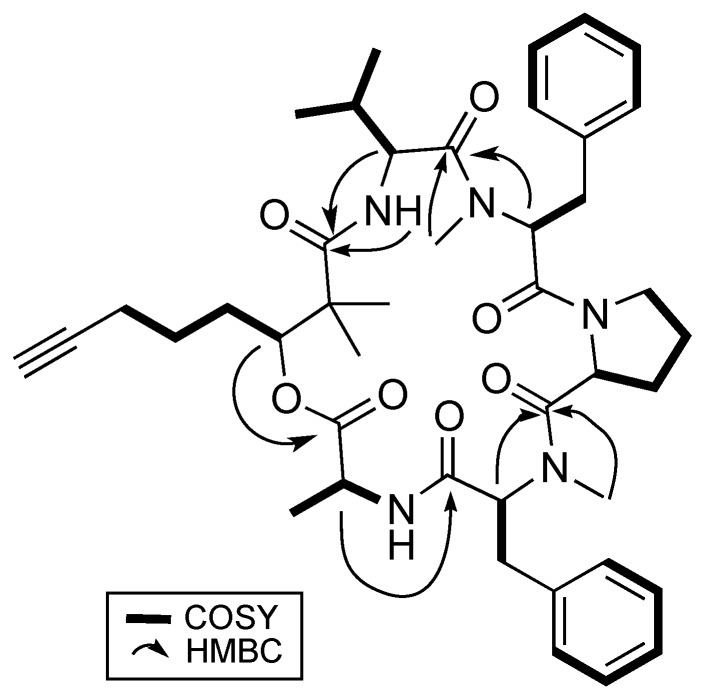
Key 2D NMR correlations of benderamide A (**1**).

Analysis based on distortionless enhancement by polarization transfer (DEPT) and 2D NMR spectral data, that included COSY, HSQC, and HMBC, revealed that **1** consisted of a non-amino acid moiety. HSQC data showed a relatively deshielded methine proton (δ_H_ 5.20) attached to a carbon associated with an oxygen atom (δ_C_ 77.4), thereby confirming the presence of an ester group, making **1** a depsipeptide. Investigation of the COSY and HSQC spectral data revealed the methine proton at H-3 has successive correlations with protons/carbons belonging to a linear carbon chain of six carbon atoms having a terminal alkyne functional group. Optimized two- and three-bond HMBC experiments confirmed the correlations of protons and carbons (Figure 2) and eventually supported the attachment of the two methyl groups at δ_H_ 1.27 and 1.19 to the quaternary α carbon, C-2. This concluded the identity of this non-amino acid moiety to be the β-hydroxy acid, 2,2-dimethyl-3-hydroxy-7-octynoic acid (Dhoya).

To determine the identity of five amino acids residues in **1**, analyses were performed on the side chain of each amino acid based on 2D NMR spectral data. The identities for four of the amino acids showed the presence of a methyl (Ala), an isopropyl (Val), and two benzyl groups (Phe) based on ^1^H-^1^H COSY data. Both benzyl-containing amino acids were also found to be *N*-methylated as observed from strong correlations of a methyl group (δ_H_ 3.59 and 2.79) to each of the α carbon C-17 and C-32, respectively, in the HMBC spectral data. HSQC and HMBC data pertaining to the last amino acid residue suggested a cyclic side group that contains a carbonyl carbon, a methine, and three methylene groups having diastereotopic protons. In addition, the unusual highly shielded ^1^H multiplet of negative chemical signal δ_H_ −0.02 to −0.10 is reminiscent of inner nitrogen-bound protons of porphyrins, was also observed. This led us to confirm the last amino acid residue to be Pro and consequently accounted for an additional degree of unsaturation in **1**. Strong HMBC correlations led to the determination of sequence of the Dhoya and the five amino acid residues to be [(*N*-Me-Phe-1)-Val-Dhoya-Ala-(*N*-Me-Phe-2)-Pro]. Weak correlations of both H-27 and H-30b to C-16 prevented the unambiguous connectivity of Pro to *N*-Me-Phe-1. This observation was also encountered by Gerwick’s group during structural elucidation of other proline-containing metabolites such as viequeamide A and veraguamides [2,10]. Considering the difference of the molecular formula based on high-resolution mass spectroscopy (HRMS) and to account for the last degree of unsaturation, **1** must possess a cyclic structure. Hence, it is imperative for **1** be identified as cyclo-[*N*-Me-Phe-1-Val-Dhoya-Ala-*N*-Me-Phe-2-Pro].

Stereoanalysis of the amino acid units in **1** was carried out based on Advanced Marfey’s analysis [11,12]. Both the *t*_R_ (min) and *m*/*z* values of the respective deprotonated adduct [M − H]^−^, were recorded for D-and L-amino acid standards derivatized with the chiral derivatizing agent, 1-fluro-2,4-dinitro-phenyl-5-L-valine amide (L-FDVA), on LC–MS system. These values were then compared with the hydrolysate of **1** derivatized with L-FDVA to reveal the presence of only L-configuration amino acid residues, i.e., L-Ala, *N*-Me-L-Phe, L-Pro, and L-Val.

**Figure 3:**
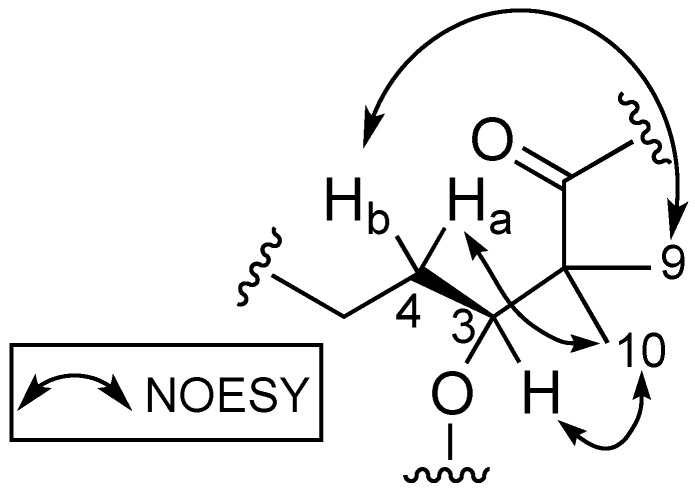
Proton correlations deduced from NOESY spectrum of benderamide A (**1**).

Stereochemical determination of the stereogenic C-3 present in the Dhoya moiety of compounds within the kulolide superfamily proved to be more challenging. Several strategies were proposed and adopted by different groups to determine the absolute configuration of this moiety present in other members of the kulolide superfamily. One such strategy is the comparison of nuclear overhauser effect (NOE) correlations data of known compounds to establish the absolute configuration of the same moiety in newly isolated compounds. To illustrate this, NOE data obtained from the known compound pitipeptolide A (**3**) was used to determine the absolute configuration of the Dhoya present in another related compound, cocosamide B (**2**) [5]. Compound **3** was determined previously to contain the 3(*S*)-Dhoya moiety [13]. It was reported that the NOESY spectral data of **3** revealed strong correlations between four different sets of protons: the methine proton, H-3 (δ_H_ 4.94), and the methyl protons at H_3_-10 (δ_H_ 1.15); H-4a (δ_H_ 1.80), and H_3_-10; one of the methylene protons H-4b (δ_H_ 1.58) and the methyl protons at H_3_-9 (δ_H_ 1.29); the methyl protons H_3_-9 and the alpha proton of L-Val (δ_H_ 6.08). In addition, no correlations were observed between H-3 and H_3_-9. The spatial protons arrangement, deduced from the NOESY spectrum of **3**, illustrates the close proximity of H-3, H-4a, and H_3_-10. Similar proton correlations, obtained from the NOESY data of **1** were also observed (Figure 3). Hence, the same 3(*S*) configuration for the Dhoya moiety in **1** was being deduced. In addition, data obtained from 1D-NOE on **1** upon selective irradiation of the methine H-3 (δ_H_ 5.20) revealed significant enhancement to the proton signals of the methylene H-4a and methyl H_3_-10, thereby indicating close proximity of these three sets of protons.

### 2.2. LC–MS/MS Molecular Networking of Benderamide A-Containing VFC Fraction

**Figure 4:**
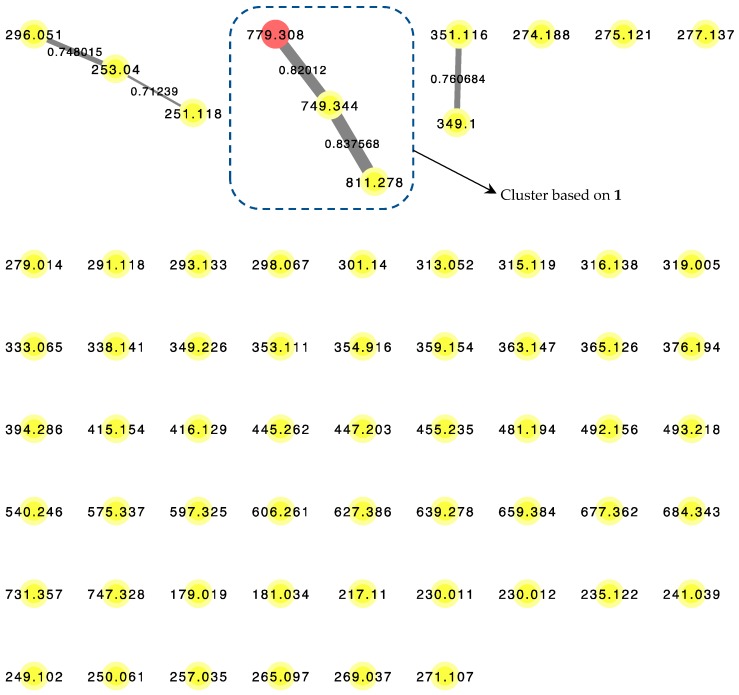
LC–MS/MS-derived molecular network of VFC-derived fraction G. Red node (*m*/*z* = 779.308) corresponds to the pseudomolecular ion of **1**. Edge thickness correspond to labeled cosine similarity between nodes.

LC–MS/MS-based Molecular Network algorithm in recent years has proven to be a useful analytical strategy in natural product research not only for dereplication, but also to detect novel chemical entities such as the cyclic octapeptide, samoamide A [14]. A posteriori approach was adopted where metabolomics data were obtained from the benderamide A-containing vacuum flash chromatography (VFC) fraction to generate a molecular network (Figure 4) to detect related analogs. Related compounds, based on MS/MS fragmentations, are displayed and identified as nodes within a cluster. The relative relatedness of the nodes is indicated by edges, with edge thickness corresponding to cosine similarity. A cluster was identified based on the node of the precursor ion with *m*/*z* 779.308, an adduct of +23 Da compared to the observed high-resolution electrospray ionization mass spectroscopy (HRESIMS) of **1** for [M + H]^+^ *m*/*z* 756.4327. The monovalent nature of this node could suggests the formula of this adduct to be [M + H + CH_3_CN − H_2_O]^+^. Such an adduct could arise due to the solvents used as the mobile phase during MS/MS analysis [15]. The identity of the node was confirmed by comparison of MS/MS spectrum obtained from this analysis and HRESIMS determination of **1**. Within this cluster, additional compounds related to **1** were observed based on *m*/*z* 749.344 and 811.278. Unfortunately, the detection or isolation of these related compounds was unsuccessfully, possibly due to insufficient quantities produced by the microorganism. The presence of these structurally related compounds highlights the versatility of the biosynthetic gene systems responsible for not only the biosynthesis of **1**, but possibly the numerous compounds in the kulolide superfamily.

### 2.3. Biological Activity of Benderamide A (**1**)

Benderamide A is a new Dhoya-containing metabolite belonging to the ‘kulolide superfamily’. The nuances in the various chemical structures of these metabolites produced by the filamentous marine cyanobacteria suggest the versatility of the inherent biosynthetic gene systems that gave rise to different bioactivities. While all members within this superfamily contain the polyketide synthase (PKS)-derived Dhoya moiety, each member is a different biosynthetic product containing different amino acid residues. A literature search on other members of the superfamily revealed that certain molecules, such as dudawalamide A [6] and kohamamide A [16] contained seven residues, while wewakpeptins A and C [17] consisted of nine resides. This could be due to possible additional modules in the cyanobacterial non-ribosomal polypeptide synthetase (NRPS) gene clusters, whereas cocosamide B [5], a compound containing only six residues, could arise from a deletion of a NRPS module. The possible promiscuity in NRPS modules may account for the formation of **1**, where Gly in **2** was substituted for Ala. Interestingly, **1** did not display antiproliferative activity against MCF7 breast or PA1 ovarian cancer cell lines when tested at 50 μM, unlike the closely related cocosamide B (**2**). **2** was reported to be cytotoxic when tested against HT-29 and MCF7 cancer cell lines with IC_50_ values of 11.0 and 39.0 μM, respectively. In addition, **1** did not show any cytotoxic activity when tested at 125 μM against the MOLT-4 cancer cell line.

Compound **1** was monitored for its effects on cell proliferation rates, metabolism, and caspase-3/-7 activation (Figure S10, Supplementary Data). PA1 cells (Panel A in Figure S10, Supplementary Data) grew more quickly than MCF7 cells (Panel B in Figure S10, Supplementary Data) but treatment with **1** did not significantly alter the growth rate over 140 h in comparison to the DMSO negative control, while cells exposed to the positive control 10 μM Nutlin displayed a lack of proliferation. Assays that measured metabolic ATP levels as an indication of viability (Panel C in Figure S10, Supplementary Data) and Caspase-3/-7 activity prior to apoptotic death (Panel D in Figure S10, Supplementary Data) also did not show any significant effect in cells treated with the natural product.

The loss in activity could be attributed to the addition of the methyl group of Ala in **1**, however, **1** and **2** should be compared within the same experimental system. Further mechanistic studies focusing on this structural feature may provide insights on the various bioactivities of Dhoya-containing members of the ‘kulolide superfamily’.

## 3. Materials and Methods

### 3.1. General Experimental Procedures

Optical rotations were measured on a Bellingham Stanley ADP 400 Polarimeter. UV and IR readings were measured on a PerkinElmer UV-Visible spectrophotometer and a PerkinElmer spectrum 100 FT-IR spectrophotometer, respectively. ^1^H, ^13^C, and 2D NMR spectra, including COSY, HSQC and HMBC, were recorded in CDCl_3_ on a 400 MHz Bruker NMR spectrometer using the residual solvent signal (δ_H_ 7.26 and δ_C_ 77.4) as internal standards. Additional 1D selective NOE and NOESY spectra were obtained on a 600 MHz Bruker NMR spectrometer using the residual solvent signal (δ_H_ 7.26 and δ_C_ 77.4) as internal standards. HPLC isolation and purification of **1** was conducted on a Shimadzu LC-8A preparative LC coupled to a Shimadzu SPD-M10A VP diode array detector. Both HRESIMS data and LC-HRMS/MS analyses were obtained on a Waters Xevo G2-XS qTOF with an ESI positive ion mode and data-dependent acquisition mode. An Agilent 1100 series coupled with an Agilent LC/MSD (Liquid Chromatography/Mass Selective Detector) trap XCT mass spectrometer, equipped with an ESI interface system in negative mode, was used for the detection of the L-Marfey’s-derivatized L/D-valine, proline, *N*-methylphenylalanine, and alanine moieties from benderamide A.

### 3.2. Biological Material

Approximately 10.0 L of the filamentous benthic marine cyanobacterium cf. *Lyngbya* sp. was collected by hand from the intertidal shores of St. John’s Island, Singapore during low tide on 24 June 2016, and stored in 70% aqueous EtOH at −20 °C before extraction. A voucher specimen on this microalga is maintained at the National Institute of Education, Nanyang Technological University, Singapore, under the code TLT/SJI/001.

### 3.3. Extraction and Isolation

Extraction of cf. *Lyngbya* sp. was carried out exhaustively using CH_2_Cl_2_/MeOH (2:1) to produce approximately 10.1 g of crude organic extract. The extract was then subjected to vacuum flash chromatography (VFC) on normal-phase Si using a combination of hexanes, EtOAc, and MeOH of increasing polarity. Nine fractions were obtained, and solvents were removed in vacuo using a rotary evaporator before being stored dried in four dram vials. All fractions were assayed at 100 ppm in the brine shrimp (*Artemia salina*) toxicity assay.

Fraction **G** (92.6% toxicity at 100 ppm in the brine shrimp toxicity assay, 266.6 mg) obtained from VFC of the organic extract was subjected to solid-phase fractionation on a Sep-Pak C_18_ cartridge (Phenomenex, Torrance, CA, USA) using 90% aqueous MeOH to remove pigments. The Sep-Pak filtrate of the brine shrimp active subfraction was subjected to semipreparative HPLC separation (Phenomenex Luna 5 μm C18, 250 × 10 mm, 85% MeOH/H_2_O in 35 min at 3.0 mL/min, detected at 210 nm) to yield semipure benderamide A (19.2 mg, *t*_R_ = 10.9 min). The final purification of the semipure benderamide A was achieved through semipreparative HPLC (Phenomenex Kinetex 5 μm Phenyl-Hexyl 250 × 4.6 mm, 50% CH_3_CN/H_2_O in 15 min at 1.2 mL/min, detected at 210 nm) to yield pure benderamide A (**1**, 3.3 mg, *t*_R_ = 10.9 min).

**Benderamide A** (**1**)**:** white, amorphous powder; [α]${}_{D}^{25}$ −98.2 (*c* 0.16, MeOH); UV (MeOH) λ_max_ (log ε*)* 215 (5.51), 286 (5.64) nm; IR (neat) ν_max_ 3482, 2816, 1657, 1618, 1569, 1012 cm^–1^; ^1^H NMR (400.13 MHz, CDCl_3_), ^13^C NMR (100.62 MHz, CDCl_3_), COSY and HMBC data, see Table 1 and Supplementary Data; HSQC, 1D selective NOE (irradiation at 5.20 ppm) and NOESY spectra, see Supplementary Data; (+)-HRESIMS *m*/*z* 756.4327 [M + H]^+^ (calcd for C_43_H_58_N_5_O_7_, 756.4336).

### 3.4. Advanced Marfey’s Analysis of Amino Acids

Hydrolysis of benderamide A (**1**, 200 μg) was achieved in 1.0 mL of 6 N HCl placed in a sealed reaction vial purged with N_2_ gas at 110 °C for 18 h. Trace HCl was removed in vacuo and the resulting hydrolysate redissolved in 0.2 mL of H_2_O. A 1% solution of L-FDVA (1000 μL) in acetone and 1 N NaHCO_3_ (50 μL) were then added to the aqueous hydrolysate and the mixture subsequently heated at 40 °C overnight. Both the increased amount of the chiral derivatizing agent used and heating time were to ensure derivatization of the sterically hindered *N*-Me-Phe. Once cooled to rt, the reaction mixtures were sequentially quenched with 2 N HCl (50 μL), then dried under vacuum and finally resuspended in CH_3_CN for LC-MS analysis. Each LC-MS analysis was carried out using a Phenomenex Kinetex C18 column (100 × 4.6 mm, 2.6 μm) and a gradient elution of H_2_O acidified with 0.1% HCOOH (A) and CH_3_CN (B) at a 0.2 mL/min flow rate. Gradient program was set to begin with 20% to 100% B for 45 min, then hold at 100% B for 5 min before reconditioning back to the starting composition in 5 min. Detection was optimized at 340 nm and mass range of *m*/*z* 320 to 500 to eliminate detection of unreacted L-FDVA. The retention times (L-configuration/D-configuration) and ESIMS deprotonated adduct ions (*t*_RL_/*t*_RD_ in min, *m*/*z* [M − H]^−^) of the L-FDVA monoderivatized amino standards were: Val (25.3/31.2, 396.4), *N*-Me-Phe (28.9/29.8, 458.5), Pro (20.5/23.4, 394.4) and Ala (19.1/20.5, 368.3). The derivatized hydrolysate peaks of **1**, identified based on observed *m*/*z*, gave retention times at 25.3, 29.0, 20.5, and 19.1 min, corresponding to L-Val, *N*-Me-L-Phe, L-Pro, and L-Ala, respectively.

### 3.5. Molecular Networking

VFC fraction **G** was filtered over C_18_ SPE cartridges by application of 1.0 mg of sample and elution with 3 mL of CH_3_CN. Solvent was removed using a rotary evaporator before being redissolved in 1 mL CH_3_CN, then vortex mixing over 5 min and transferred into separate Eppendorf tubes. Tubes were then centrifuged at 10,000 rpm at 4 °C over 10 min and the supernatant was aliquoted and finally diluted with ACN to 10,000× dilution. One-and-a-half microliters of each 10,000× dilution sample was subjected to LC–HRMS/MS performed with a Waters Acquity BEH (C_18_ 50 mm × 2.1 mm, 1.7 μm) column and maintained at a column temperature of 40 °C and sample temperature at 4 °C using a step elution program of ‘1’ based on Waters system. Step elution program was as follows: mobile phase of 98% CH_3_CN in 0.1% aq. HCOOH/0.1% aq. HCOOH, run time of 14 min and flow rate of 0.5 mL/min. All the mass spectra were recorded in the positive-ion mode. Data was collected in the data-dependent acquisition mode, in which the first ten most intense ions of a full-scan mass spectrum were subjected to tandem mass spectrometry (MS/MS) analysis. MS/MS scans were obtained for selected ions with a mass range of 100 to 2000 Da, MS scan time of 0.33 s over 12 min, MS/MS scan time of 0.10 s, and a collision energy ramp of 10–50 V. The chromatogram was converted digitally to .mzXML files using freely available MSConvert software and submitted to Global Natural Product Social Molecular Networking (GNPS). A molecular network was then generated to interconnect MS/MS spectra along with blank CH_3_CN injection.

### 3.6. IncuCyte Live Cell Analysis Imaging System

Human ovarian PA1 and breast MCF7 cell lines were obtained from ATCC and maintained in DMEM supplemented with 10% FBS and 1% penicillin–streptomycin at 37 °C with 5% CO_2_. Cells were seeded in clear colorless 96 well plates (TPP) at 4000 cells per well 24 h prior to treatment with various concentrations of **1** or with racemic Nutlin-3 (444143, Merck, Darmstadt, Germany) as a positive control. Plates were placed in the IncuCyte chamber (Essen BioScience Inc., Welwyn Garden City, UK) for live cell analysis and imaging over a period of 140 h. Three images per well were collected every 2 h and the manufacturer’s software was used to determine the percentage confluence. Each condition was averaged over three wells and the data presented is representative of triplicate experiments.

### 3.7. Cell Titer-Glo and Caspase 3/7-Glo Assays

Cells were seeded in white walled 96 well plates (Greiner) at 4000 cells per well 24 h prior to treatment with various concentrations of **1** or with racemic Nutlin-3 (Merck, 444143) as a positive control. Cell Titer-Glo luminescent cell metabolism (viability) reagent (G7570, Promega, Madison, WI, USA) or Caspase-Glo -3/-7 substrate (G8090, Promega, Madison, WI, USA) was added 72 h following treatment, according to the manufacture’s protocol. Luminescent signals were determined using an EnVision 2104 Multilabel reader. Each condition was averaged over three wells and the data presented is representative of triplicate experiments.

## 4. Conclusions

The new cyclic depsipeptide, benderamide A (1), was isolated from the marine cyanobacterium cf. *Lyngbya* sp., collected from St. John’s Island, Singapore. Data obtained from various NMR spectroscopic and Marfey’s analysis provided the complete molecular structure of 1, making this Dhoya-containing metabolite a new member of the ‘kuololide superfamily’. Compound 1 did not display antiproliferative activity when tested against the MCF-7 breast and PA1 ovarian cancer cell lines despite having a slight structural difference with the cytotoxic analog, cocosamide B (2). More importantly, its antiproliferative inactivity against MCF-7 breast cancer cell line postulate the importance of the methyl group in Ala as a structural feature in future mechanistic studies.

## Figures and Tables

**Table 1:** 1D NMR spectroscopic data for benderamide A (**1**) in CDCl_3_ (^1^H 400 MHz, ^13^C 100 MHz).

Unit	C/H No.	δ_C_, Multi.	δ_H_ (*J* in Hz)	COSY	HMBC *^a^*
Dhoya	1	176.6, C			
	2	146.1, C			
	3	177.4, CH	5.20, dd (11.0, 2.0)	H-4a, H-4b	1, 2, 4, 5, 9, 41
	4a	127.9, CH_2_	1.73–1.78, m	H-4b, H-5	2, 5, 6
	4b		1.54, d (6.9)	H-3, H-4a	5
	5	124.7, CH_2_	1.49–1.44, m	H-4a, H-6	3, 4, 6, 7
	6	118.3, CH_2_	2.21, td (6.7, 2.6)	H-5	4, 5, 7, 8
	7	183.7, C			
	8	169.3, CH	1.95, t (2.5)		6
	9	117.7, CH_3_	1.27, br. s		1, 2, 3
	10	123.6, CH_3_	1.19, br. s		1, 2, 3
Val	11	172.3, C			
	12	155.7, CH	4.28, dd (9.5, 7.5)	NH, H-13	1, 11, 14, 15
	13	130.2, CH	1.91–1.85, m	H-12, H-14, H-15	12, 15
	14	119.5, CH_3_	0.98, d (6.7)	H-13	12, 13, 15
	15	118.8, CH_3_	0.92, d (6.7)	H-13	12, 13, 14
	*N*H		5.70, d (7.2)	H-12	1, 12
*N*-Me-Phe-1	16	169.2, C			
	17	154.4, CH	5.06, dd (12.1, 3.4)	H-18a, H-18b	11, 16, 18, 19, 25
	18a	138.0, CH_2_	3.17, s	H-17, H-18b	16, 17, 19, 20, 24
	18b		3.00–2.95, m	H-17	16, 17, 19, 20, 24
	19	137.8, C			
	20/24	130.1, CH	7.42, m	H-21/23	18, 21, 22, 23
	21/23	129.0, CH	7.22–7.19, m	H-20/24, H-22	19, 20, 21, 23, 24
	22	127.2, CH	7.19–7.14, m	H-21/23	20, 21, 23, 24
	25	132.4, CH_3_	3.59, s		11, 17
Pro	26	171.7, C			
	27	156.4, CH	3.15–3.09, m	H-28a	28, 29
	28a	129.9, CH_2_	−0.02–(−0.10), m	H-28a, H-29a	26, 27
	28b		0.55–0.47, m	H-28b	26, 27
	29a	122.3, CH_2_	0.87–0.83, m	H-29a	27, 28
	29b		1.19, br. s	H-27, H-28a, H-28b, H-30a, H-30b	30
	30a	146.4, CH_2_	3.23–3.19, m	H-29a	29
	30b		3.40–3.33, m	H-29a	28, 29
*N*-Me-Phe-2	31	169.9, C			
	32	163.4, CH	3.95, dd (9.0, 4.4)	H-33a, H-33b	26, 31, 33, 34, 40
	33a	135.4, CH_2_	2.75–2.68, m	H-32, H-33a	31, 32, 34, 35, 39
	33b		3.63, d (4.0)	H-32	31, 32, 34, 35, 39
	34	138.4, C			
	35/39	129.8, CH	7.06, m	H-36/38	33, 36, 37, 38
	36/38	128.7, CH	7.22–7.19, m	H-35/39	34, 36, 38
	37	127.2, CH	7.19–7.14, m	H-37	35, 36, 38, 39
	40	131.1, CH_3_	2.79, s		26, 32
Ala	41	168.9, C			
	42	148.8, CH	4.99, dd (9.0, 7.0)	NH, H-43	31, 41, 43
	43	118.4, CH_3_	1.54, d (6.9)	H-42	41, 42
	*N*H		9.03, d (9.1)	H-42	31, 32, 41, 42, 43

*^a^* HMBC correlations, optimized for ^2/3^*J*_CH_ = 8.0 Hz, are from proton(s) stated to the indicated carbon.

## Supplementary Materials

The following are available online at http://www.mdpi.com/1660-3397/16/11/409/s1, morphology of cyanobacteria (TLT/SJI/001) observed under microscope, isolation flowchart, 1D and 2D NMR, and cytotoxicity results of **1**.
