# Supplementary material for: Benderamide A, a Cyclic Depsipeptide from a Singapore Collection of Marine Cyanobacterium cf. Lyngbya sp
Source: Mar Drugs. 2018 Oct 26;16(11):409. doi: 10.3390/md16110409 (PMC6266194; doi:10.3390/md16110409)
Supplement: Supplementary file 1 [file marinedrugs-16-00409-s001.pdf]

Benderamide A, a Cyclic Depsipeptide from a Singapore Collection of Marine Cyanobacterium *Lyngbya* sp.

Chi Ying Gary Ding[1], Ji Fa Marshall Ong[1], Hui Chin Goh[2], Cynthia R. Coffill[2], Lik Tong Tan[1,*]

[1] Natural Sciences and Science Education, National Institute of Education, Nanyang Technological University, 1 Nanyang Walk, Singapore 637616, Singapore

[2] p53 Laboratory, Agency for Science, Technology and Research (A*STAR), #06-06, Immunos, 8A Biomedical Grove, Singapore 138648, Singapore

SUPPLEMENTARY DATA

**Figures**		**Page**
Figure S1.	Morphology of TLT/SJI/001 under microscope	2
Figure S2.	Flow chart of isolation and purification of benderamide A (**1**)	3
Figure S3.	^{1}H spectrum of benderamide A (**1**) recorded in $CDCl_3$ at 400 MHz	4
Figure S4.	^{13}C spectrum of benderamide A (**1**) recorded in $CDCl_3$ at 400 MHz	5
Figure S5.	COSY spectrum of benderamide A (**1**) recorded in $CDCl_3$ at 400 MHz	6
Figure S6.	HSQC spectrum of benderamide A (**1**) recorded in $CDCl_3$ at 400 MHz	7
Figure S7.	HMBC spectrum of benderamide A (**1**) recorded in $CDCl_3$ at 400 MHz	8
Figure S8.	NOESY spectrum of benderamide A (**1**) recorded in $CDCl_3$ at 600 MHz	9
Figure S9.	1D selective NOE (irradiation at 5.20 ppm) spectrum of benderamide A (**1**) recorded in $CDCl_3$ at 600 MHz	10
Figure S10.	Cellular proliferation, metabolism and caspase determination	11

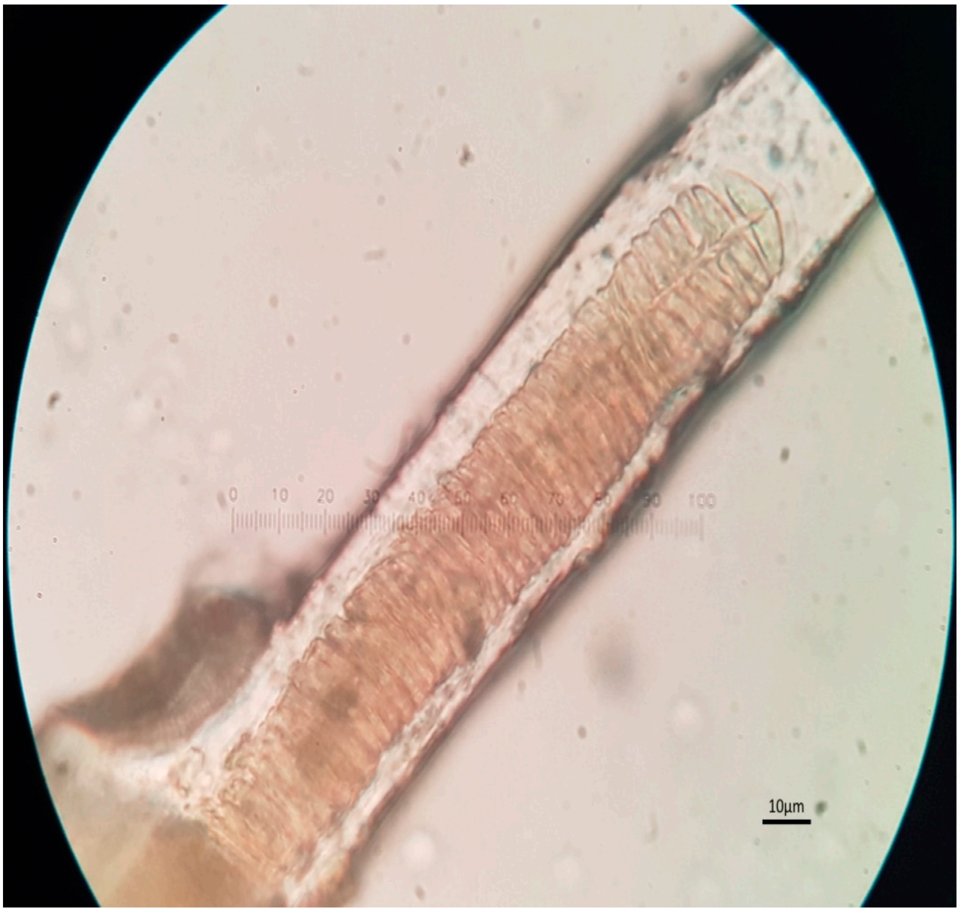

TLT/SJI/001
Lyngbya sp.,
Olympus CX31RBSF, 100x Zoom
Lens

Figure S1. Morphology of TLT/SJI/001 under microscope (viewed under 100 × zoom lens)

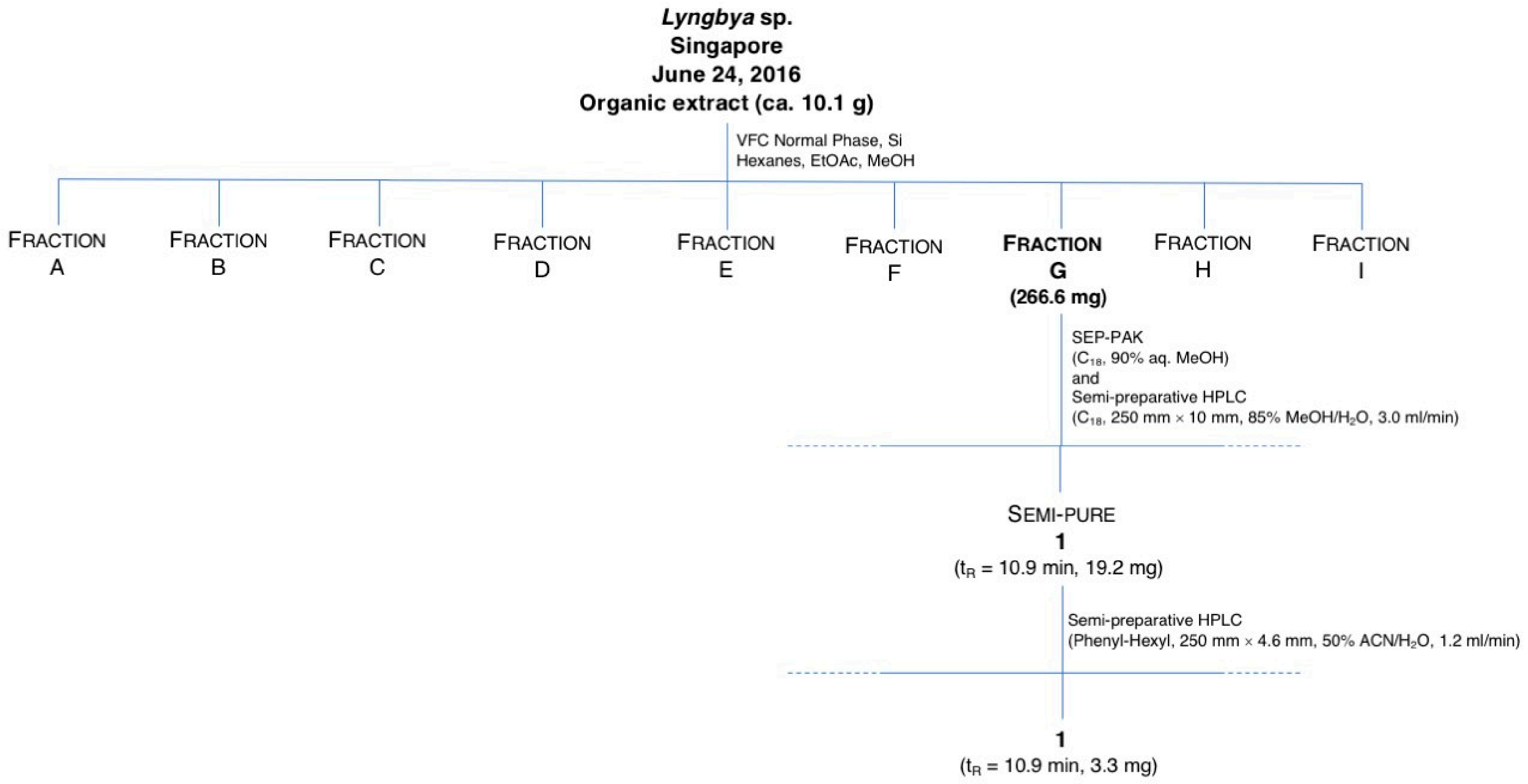

Figure S2. Flow chart of isolation and purification of benderamide A (**1**)

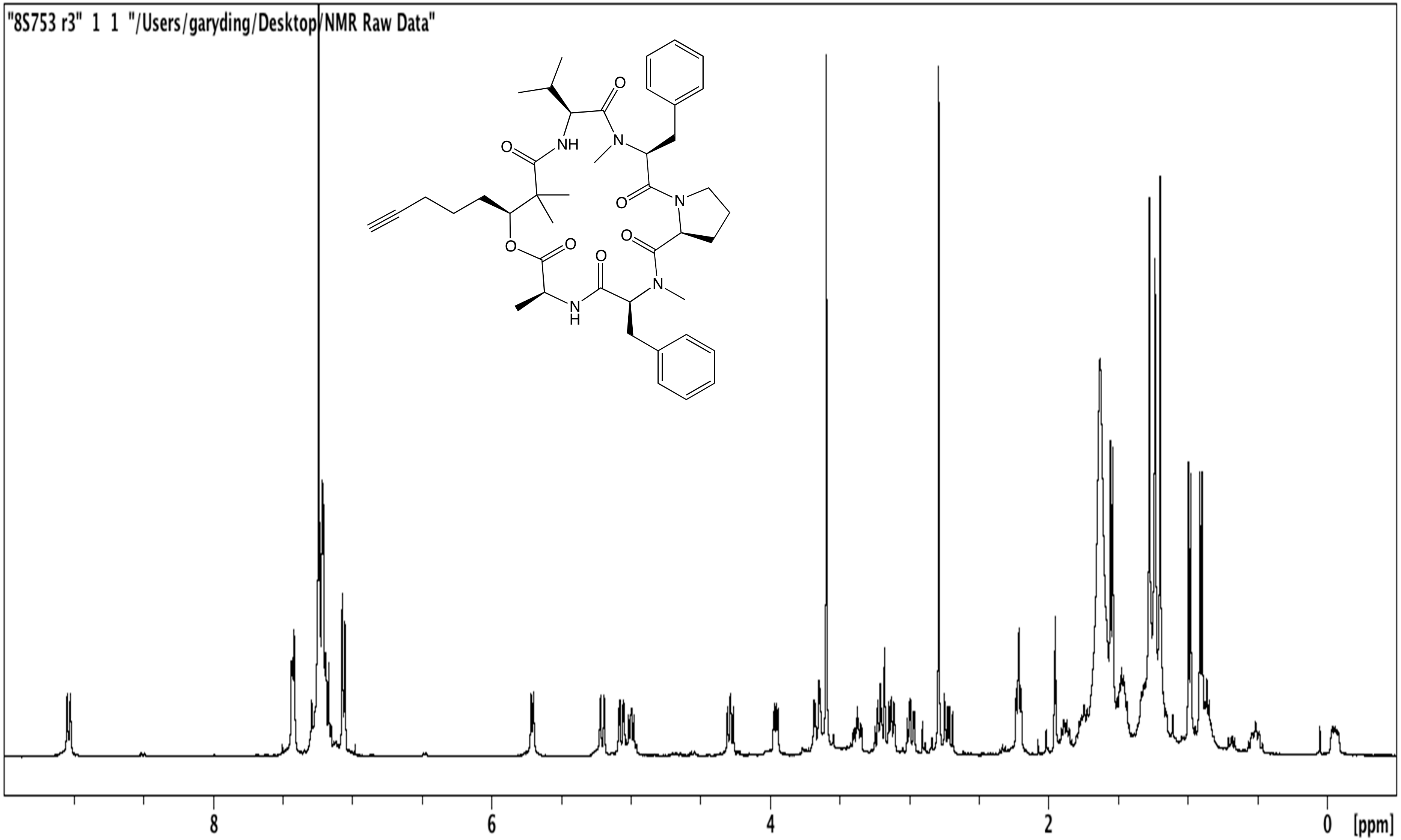

Figure S3. ^{1}H spectrum of benderamide A (**1**) recorded in $CDCl_3$ at 400 M Hz

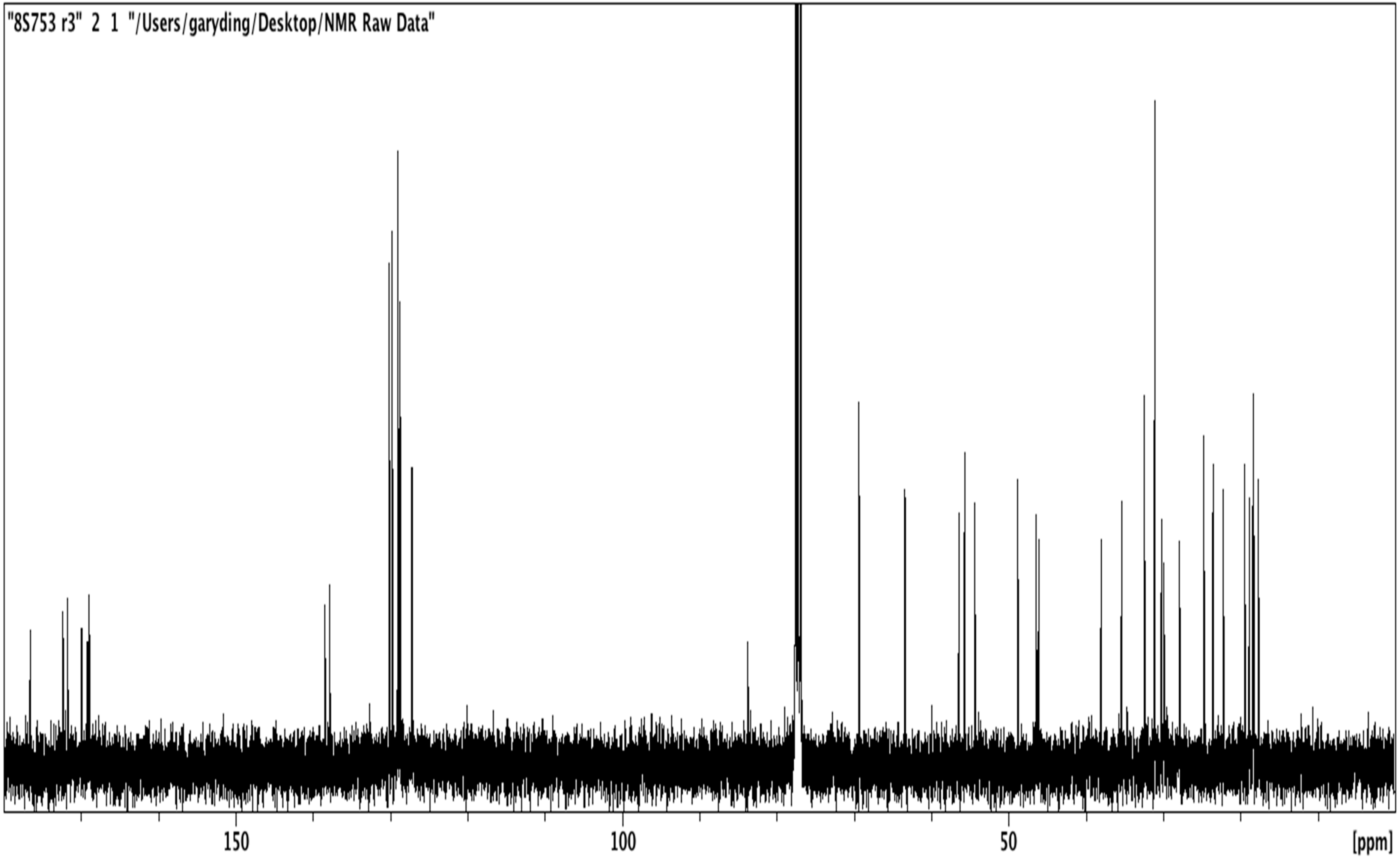

Figure S4. ^{13}C spectrum of benderamide A (**1**) recorded in $CDCl_3$ at 400 MHz

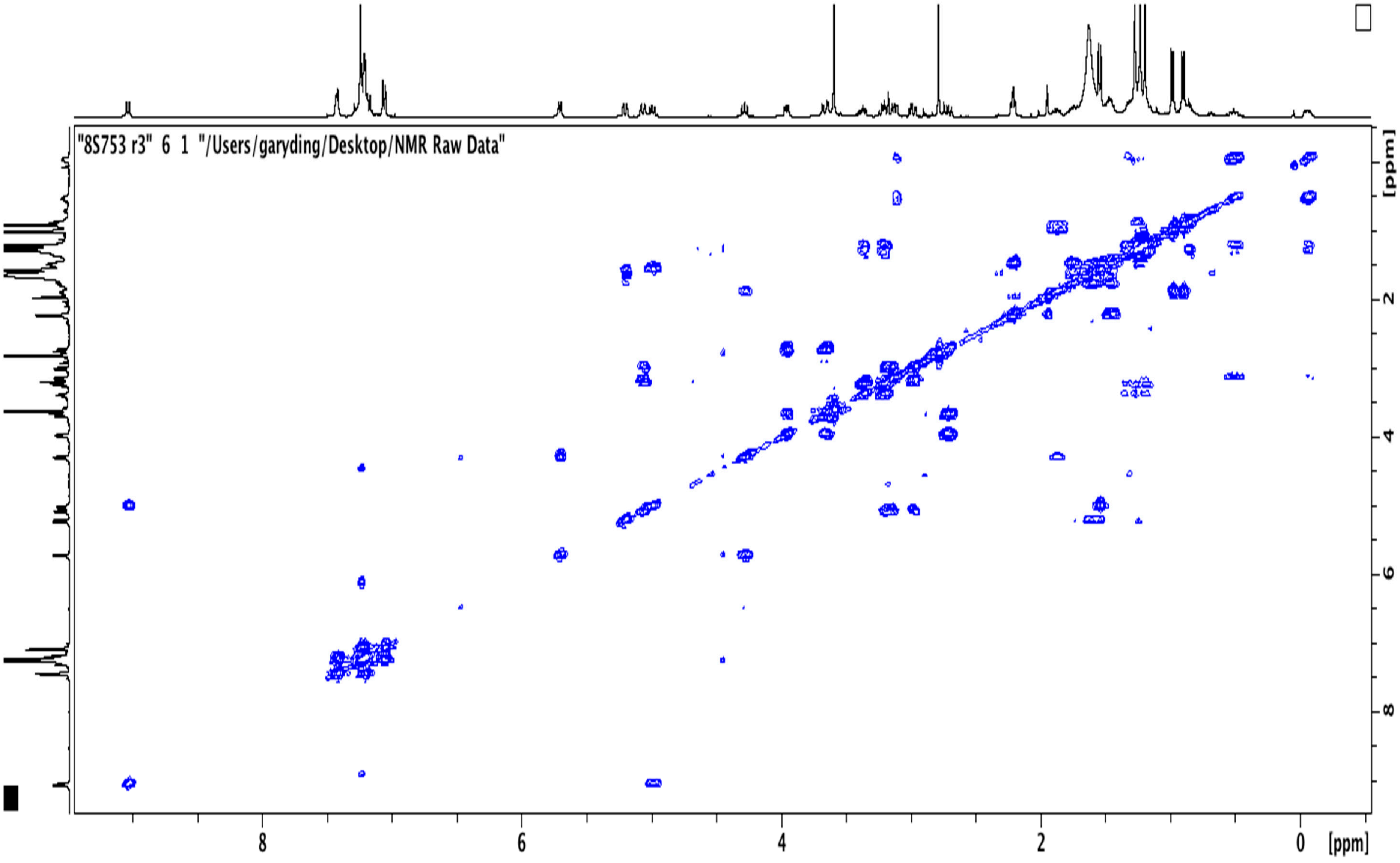

Figure S5. COSY spectrum of benderamide A (**1**) recorded in $CDCl_3$ at 400 MHz

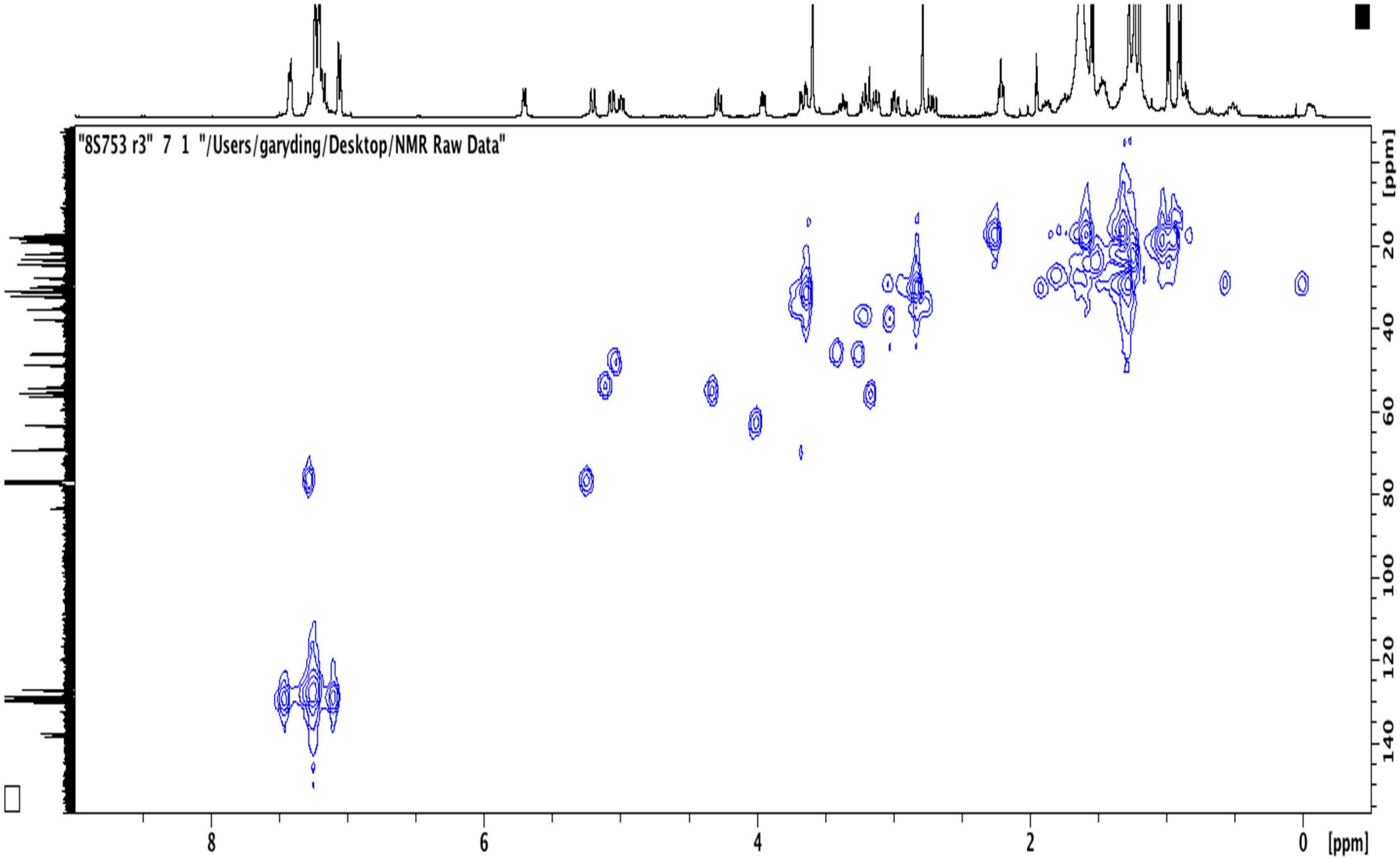

Figure S6. HSQC spectrum of benderamide A (**1**) recorded in $CDCl_3$ at 400 MHz

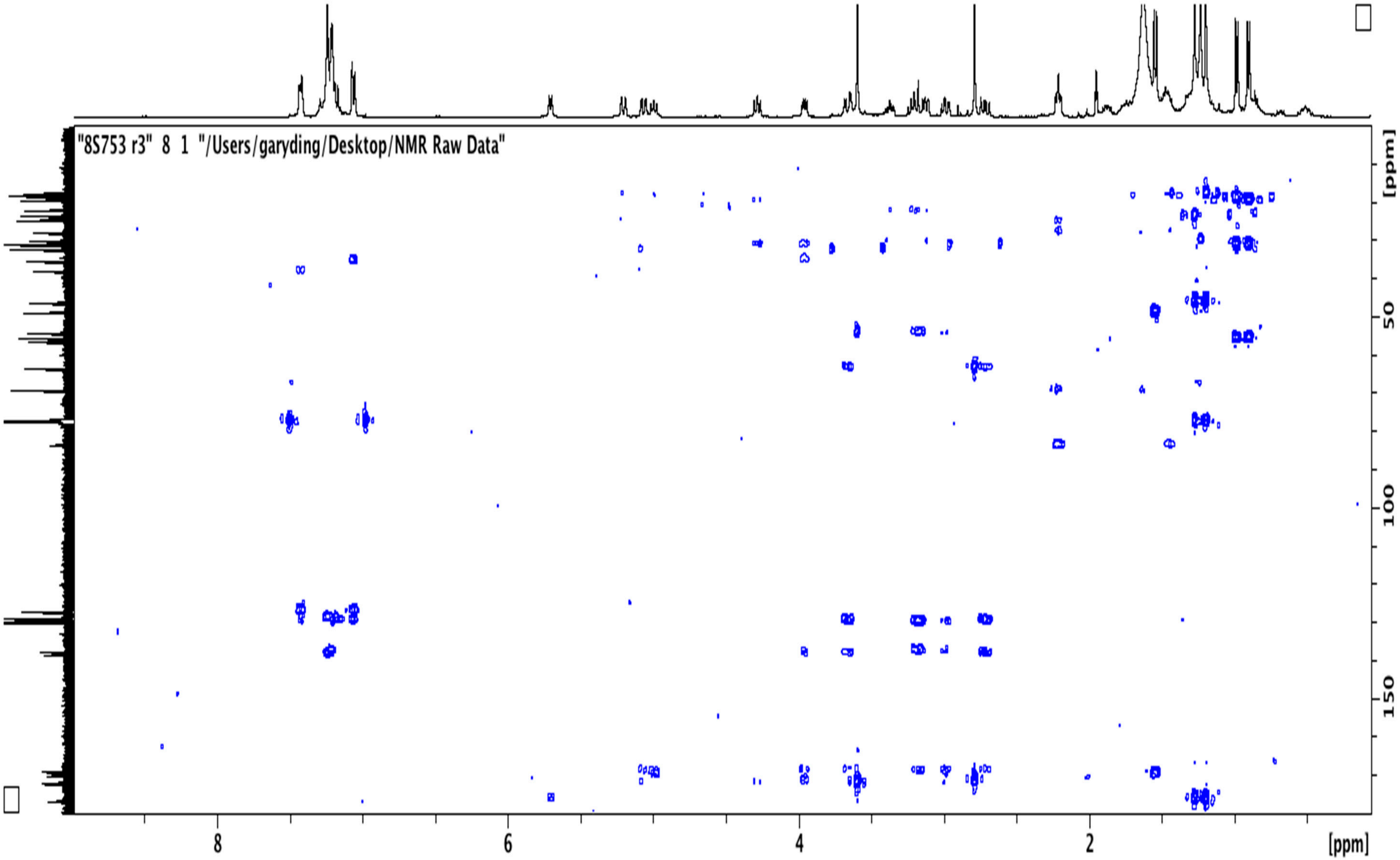

Figure S7. HMBC spectrum of benderamide A (**1**) recorded in $CDCl_3$ at 400 MHz

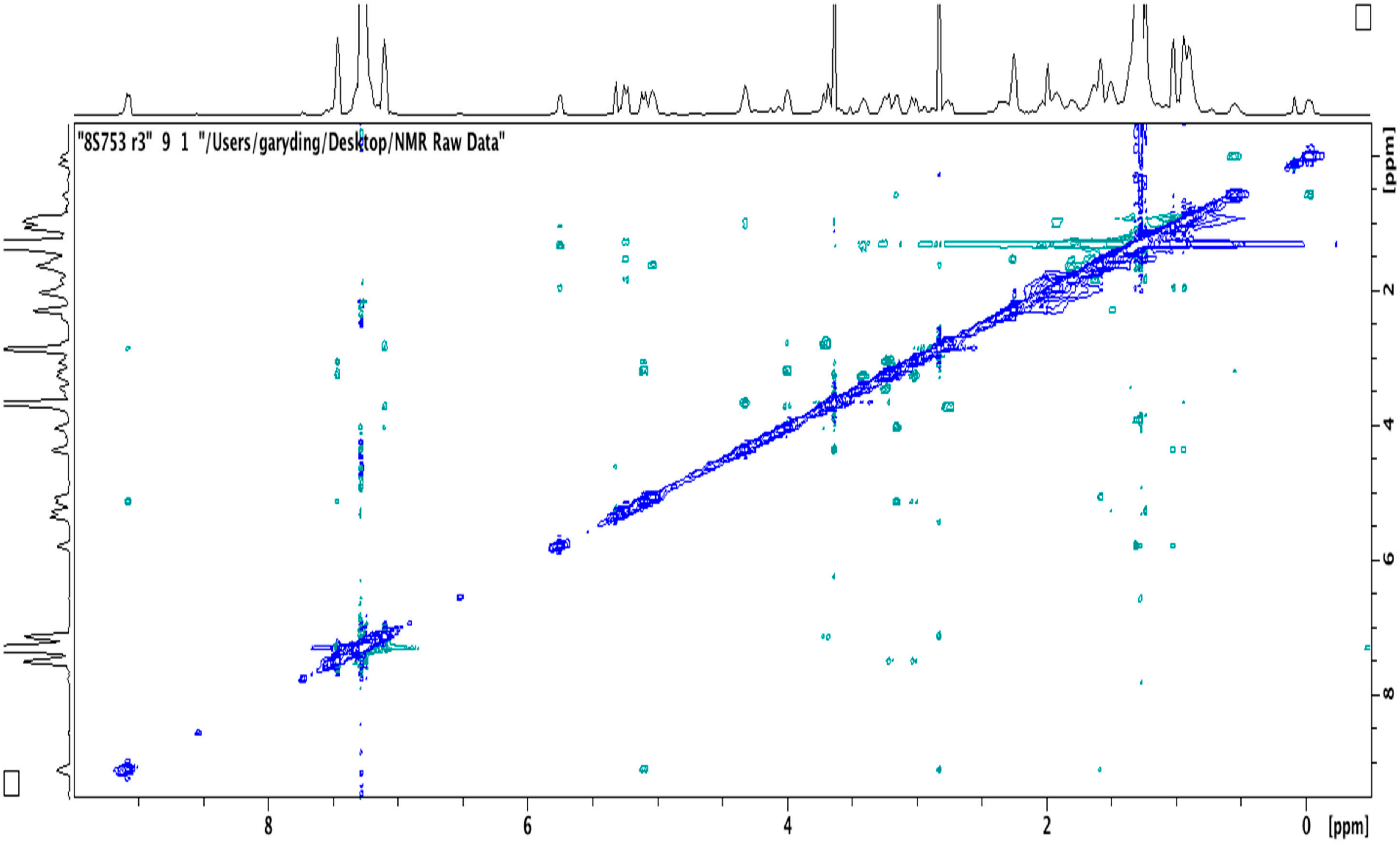

Figure S8. NOESY spectrum of benderamide A (**1**) recorded in $CDCl_3$ at 600 MHz

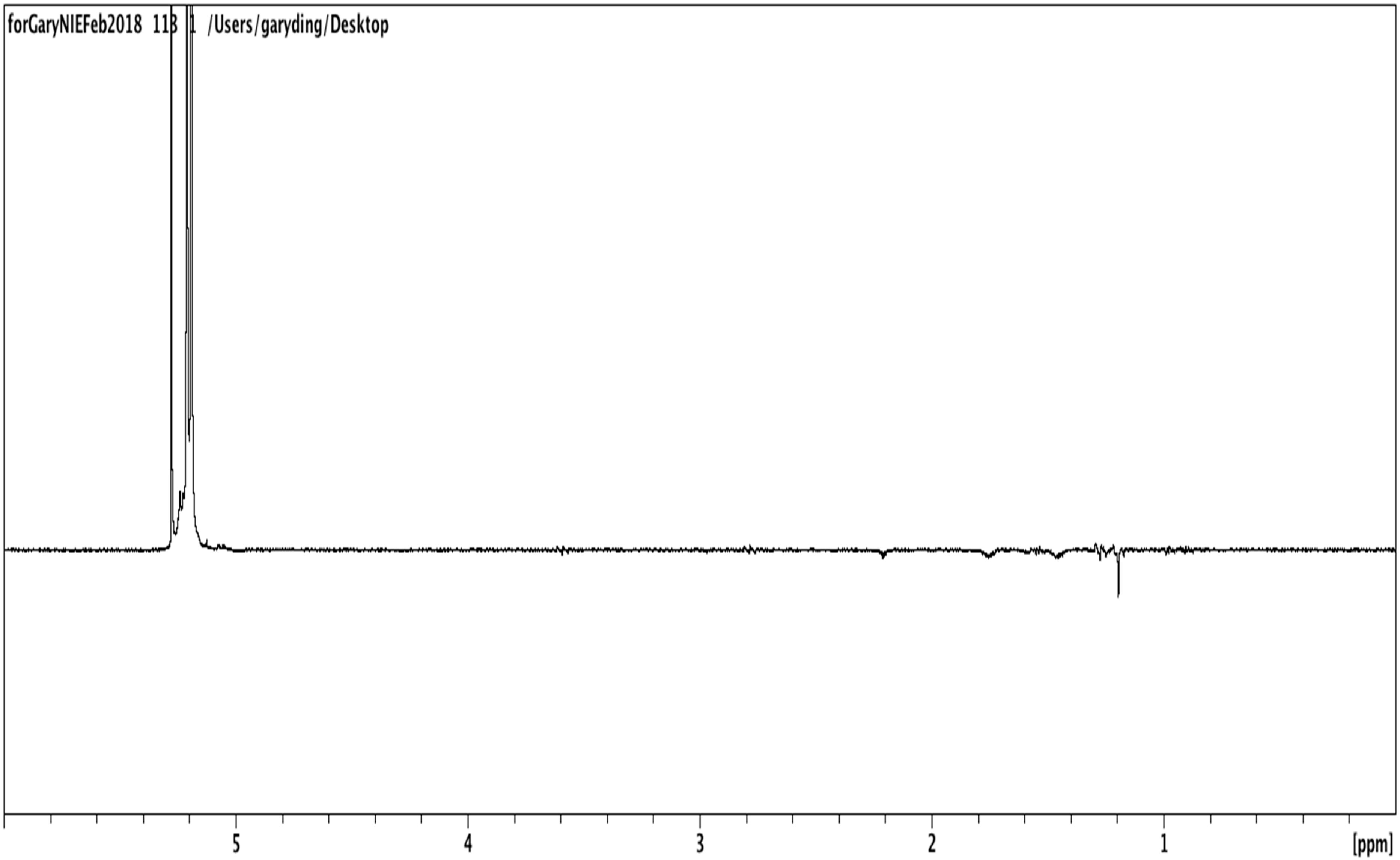

Figure S9. 1D selective NOE (irradiation at 5.20 ppm) spectrum of benderamide A (**1**) recorded in $CDCl_3$ at 600 MHz

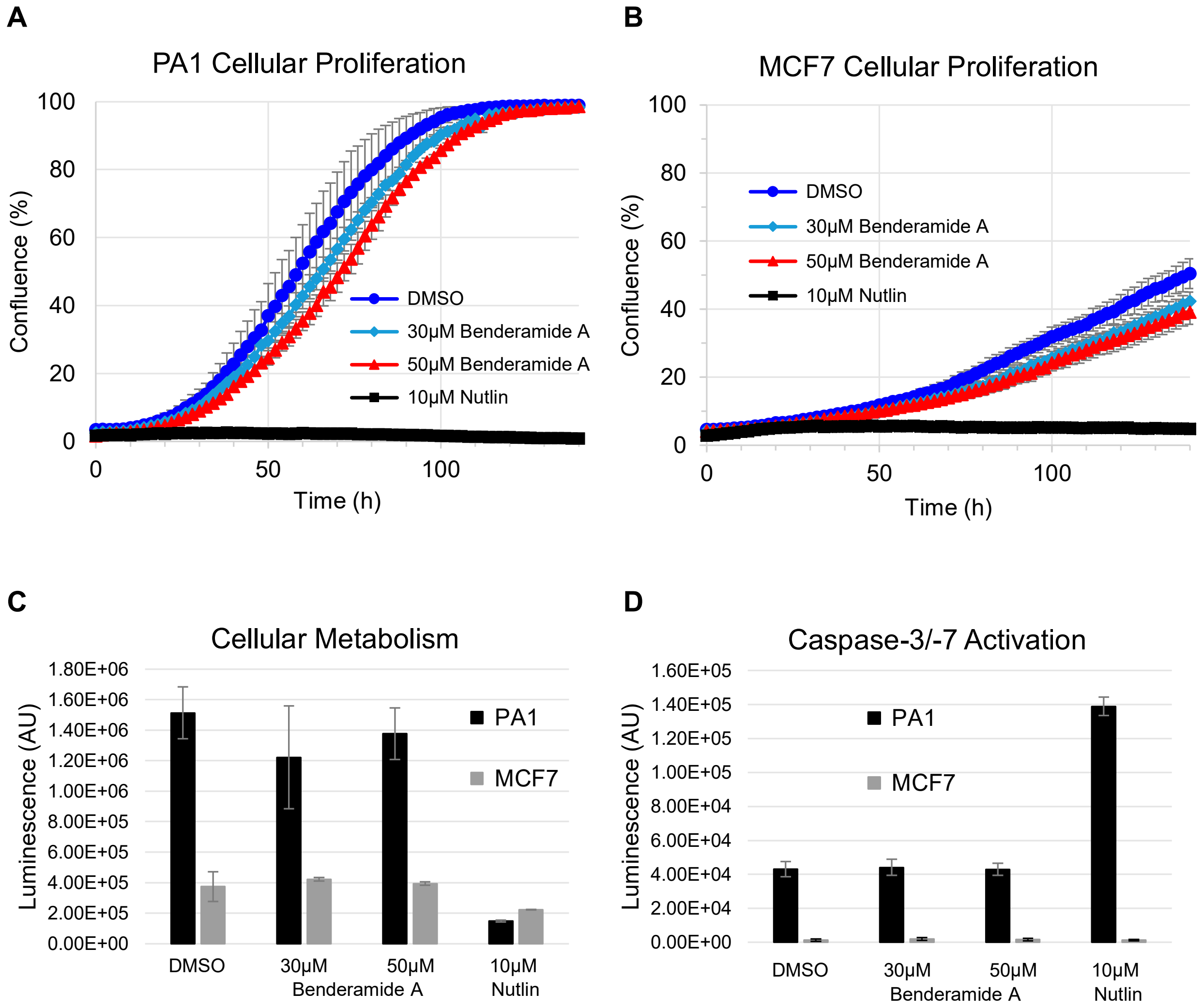

Proliferation as determined by percent confluence of human ovarian PAI (Panel A) or breast MCF7 (Panel B) cells was measured using IncuCyte for 140 h in the presence of DMSO (dark blue circles), 30 μM Benderamide A (light blue diamonds), 50 μM Benderamide A (red triangles) or 10 μM Nutlin (black squares). Cell Titre Glo metabolism (viability) assay (Panel C) or Caspase-3/-7 (Panel D) was measured for both PA1 (black bars) or MCF7 (grey bars) 72 h following the indicated treatment. Each data point is the average over three wells for each condition and the data presented is representative of triplicate experiments. Error bars represent standard error.

Figure S10. Cellular proliferation, metabolism and caspase determination
